# Multidisciplinary Approach to Rehabilitation after Tumor Resective Jaw Surgery: A 9-Year Follow-Up

**DOI:** 10.1155/2020/8867320

**Published:** 2020-12-10

**Authors:** Igor Smojver, Marko Vuletić, Spomenka Manojlović, Dragana Gabrić

**Affiliations:** ^1^St. Catherine Specialty Hospital, Zagreb, Croatia; ^2^Department of Oral Surgery, School of Dental Medicine, University of Zagreb, Croatia; ^3^Croatian Society for Hospital Dentistry / Dentistry of Special Care of the Croatian Medical Association, Zagreb, Croatia; ^4^Department of Pathology, School of Medicine, University of Zagreb, Croatia

## Abstract

A 36-year-old male patient presented at the Department of Maxillofacial Surgery, University Hospital Clinic Zagreb in December 2010 due to a swelling of the left body of the mandible that was noticed 4 months earlier. The patient was referred regarding an atypical clinical and radiological finding in the form of a multicystic appearance in the corpus of the left mandible and teeth mobility in the same region. A probatory biopsy was performed under local anesthesia and sent for histopathologic examination which reported odontogenic myxoma. The tumor was removed with a marginal resection of the mandible from the left first incisor to the left second molar. Two years after surgery, in January 2013, the patient was referred to the oral surgeons for implant-prosthodontic rehabilitation. Two narrow implants were placed at positions 32 and 36, and three months afterwards, implant-retained denture was made using locator connections to compensate lost teeth and to correct the ratio of soft tissues and facial contours. The patient was followed-up for 9 years without any functional and aesthetic problems. Loosing function and aesthetic morbidity, after radical surgical treatment, often have compromised the quality of life of this group of patients. It is important to highlight the need for multidisciplinary collaboration for the complete rehabilitation of the patient after surgical oncology of the maxillofacial region.

## 1. Introduction

Myxomas are very rare benign tumors of ectomesenchymal origin [1]. These tumors are locally invasive and can occur in various tissues, such as the heart, bones, skin, skeletal muscle, subcutaneous tissue, genitourinary tract, and aponeuroses [2, 3]. Myxomas of the head and neck region occur mainly in the jaw bones, with a very small minority occurring in the pharynx, larynx, paranasal sinuses, and other soft tissues [4]. Odontogenic myxoma, also termed as odontogenic fibromyxoma or myxofibroma, is a subtype of myxoma occurring mainly in the hard, bony tissues of the face, although the lesion may also occur in the surrounding soft tissues [5]. This neoplasm often has infiltrating and locally aggressive character, and according to the WHO, it is the third most frequent odontogenic tumor after odontoma and ameloblastoma [6, 7]. The WHO [6] reports that odontogenic myxoma is up to twice common in females while others report that there is no sex predilection [3, 8]. The tumor may be an incidental finding or may cause symptoms, including pain, paresthesia, and tooth mobility [9]. Thoma and Goldman [10] first described myxomas of odontogenic origin, on the basis of site of occurrence, by association with missing teeth, age at occurrence, and histopathological examination, which showed structural resemblance with dental mesenchyme and the sporadic presence of islands of odontogenic epithelium.

Diagnosis of odontogenic myxomas is based on radiological, histopathological, and clinical findings [11]. Radiographically, a unilocular or multilocular radiolucent image and mixed radiolucency and radio-opacity have been reported [12]. Odontogenic myxoma consisted of spindle shaped to stellate cells in an intercellular matrix rich in mucoid, with no encapsulation and sporadically scattered residual bony trabeculae [13], while the stroma may consist of collagen bundles, hence the designation of myxofibroma [14]. Clinically, it can be characterized by cortical expansion, potential to cause bone destruction, slow growth, soft tissue infiltration, tooth movement, and root resorption [2]. In the differential diagnosis odontogenic keratocyst, follicular cyst, ameloblastoma, aneurysmal bone cyst, central giant cell granuloma, and intraosseous hemangioma [15] may be included. The ideal treatment of this neoplasm has not been fully agreed in the literature. There are different treatment modalities from enucleation and curettage to en bloc resection [7, 9]. If the approach is more radical, there is a lower rate of recurrence but there are more associated morbidities, especially in the aesthetic and functional point of view [9].

The aim of the present research is to describe a case of a 36-year-old man who developed an odontogenic myxoma of the mandible 9 years ago and successful implanto-prosthodontic rehabilitated after resective jaw surgery with a multidisciplinary approach. The purpose of this case report was to highlight the need for multidisciplinary collaboration for the complete rehabilitation of the patient after surgical oncology of the maxillofacial region.

## 2. Case Presentation

A 36-year-old male patient came to the Department of Maxillofacial Surgery, University Hospital Clinic Zagreb in December 2010 due to a swelling of the left body of the mandible that was noticed 4 months earlier. The patient did not report any other symptoms. After examination, the patient was referred to a maxillofacial surgeon regarding an atypical clinical and radiological finding in the form of a multicystic appearance (bubble-like) in the corpus of the left mandible and pathological teeth mobility in the region of the swelling. There were no enlarged lymph nodes in the neck and head area. Differential diagnosis included keratocystic odontogenic tumor, ameloblastoma, central giant cell granuloma, and odontogenic myxoma.

The patient denied any systemic disease or condition and reported no previous surgeries. A general physical examination was unremarkable. The patient did not have any deleterious habits such as alcohol consumption or smoking and without previous history of swelling or trauma of the mandible. On extraoral examination, the patient displayed some facial asymmetry with an obvious firm and diffuse swelling on the left side of the mandible. The overlying skin was normal in appearance. Intraorally, there was a diffuse swelling in the buccal vestibule. Teeth in the third quadrant were without any pain sensation but with pathological mobility. Lower incisors and canine had mobility grade I and premolars and first molar grade II according to the Miller classification.

**Figure 1:**
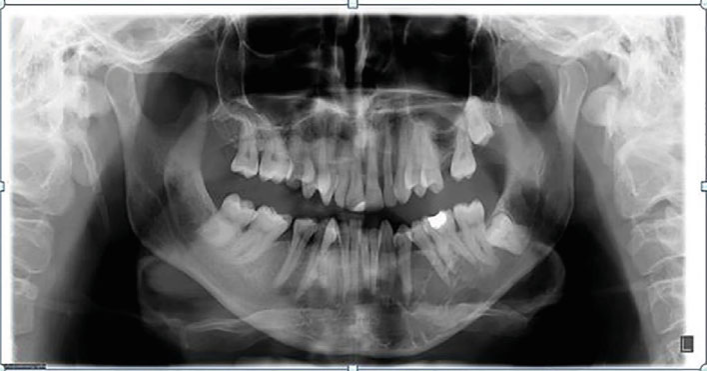
First OPG with multicystical lesion in the left mandible surrounding teeth 33 to 36 (2010).

The orthopantomogram (OPG) showed a multilocular radiolucency, with fine trabeculation, extending from the left canine, towards the first molar (Figure 1) with no root resorption. A CT scan was made afterwards and revealed the destruction of the buccal cortex of the left body of the mandible. A probatory biopsy was performed under local infiltration anesthesia (4% articaine with epinephrine 1 : 200 000; 1.8 mL), and the specimen was sent for histopathologic examination which reported odontogenic myxoma.

**Figure 2:**
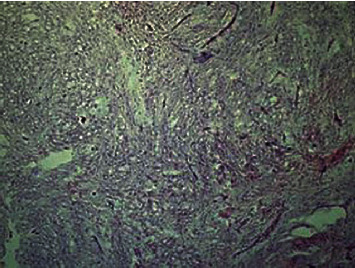
Representative photomicrograph of odontogenic myxoma (hematoxylin and eosin, original magnification 100x).

**Figure 3:**
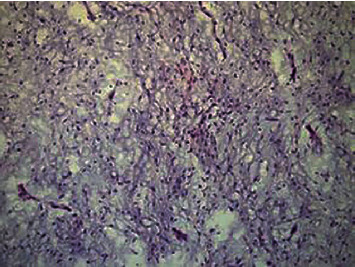
Spindle-shaped cells with long fine cytoplasmic processes, within an abundant, myxoid ground substance, containing small capillaries and fragments of bone trabecula (hematoxylin and eosin, original magnification 200x).

In accordance with the ethical protocol of the School of Dental Medicine, University of Zagreb, Croatia, written consent was obtained from the patient before surgery in general anesthesia in January 2011. Lower incisors, canine, premolars, and first molar were extracted just before the first incision. A mucoperiosteal flap with full exposition of the buccal aspect of the tumor was performed. The tumor was removed with a marginal resection of the mandible from the left first incisor to the left second molar. The surgical site was examined and precociously cleaned of all myxomatous tissue, and a reconstruction plate was used to reinforce the resected mandible. The surgical specimen was a soft, gelatinous mass measuring approximately 1 cm in diameter, with a mucinous appearance. Histologically, the tumor was composed of randomly oriented spindle-shaped cells with long fine cytoplasmic processes, within an abundant, myxoid ground substance, containing small capillaries and fragments of bone trabecula. Odontogenic epithelium was absent, and clear margins were confirmed (Figures 2 and 3). The patient recovered completely, with no postoperative paresthesia or facial asymmetry.

**Figure 4:**
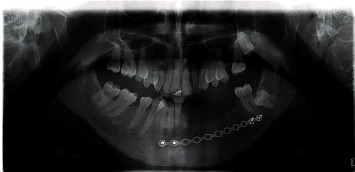
OPG 2 years after resective surgery (2013).

**Figure 5:**
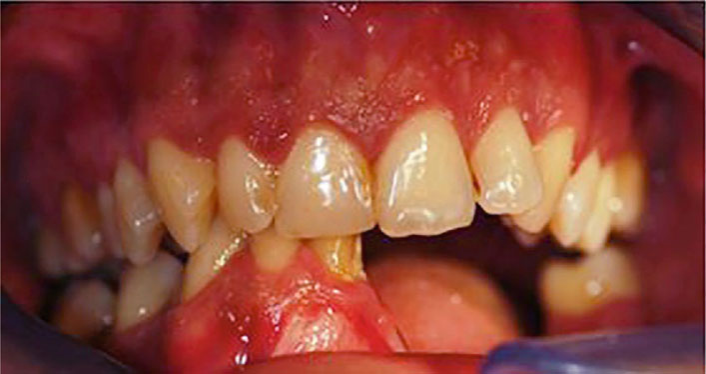
Clinical appearance after 2 years postresective surgery-frontal view.

**Figure 6:**
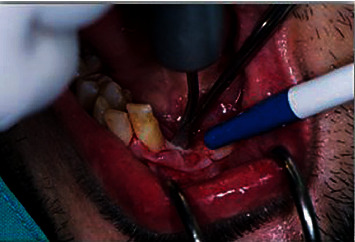
Bone splitting with piezo bone saw during implant placement.

Two years after surgery, in January 2013, the patient was referred to the Department of Oral Surgery, School of Dental Medicine, University of Zagreb for implant-prosthodontic rehabilitation. A control OPG (Figure 4) and clinical examination (Figures 5 and 6) showed complete recovery with a residual defect in the alveolar extension and left concavity.

**Figure 7:**
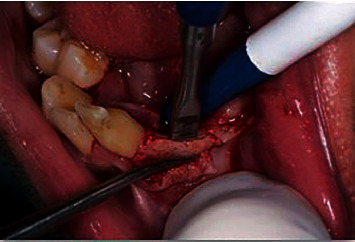
Bone splitting with bone mallet and chisel during implant placement.

**Figure 8:**
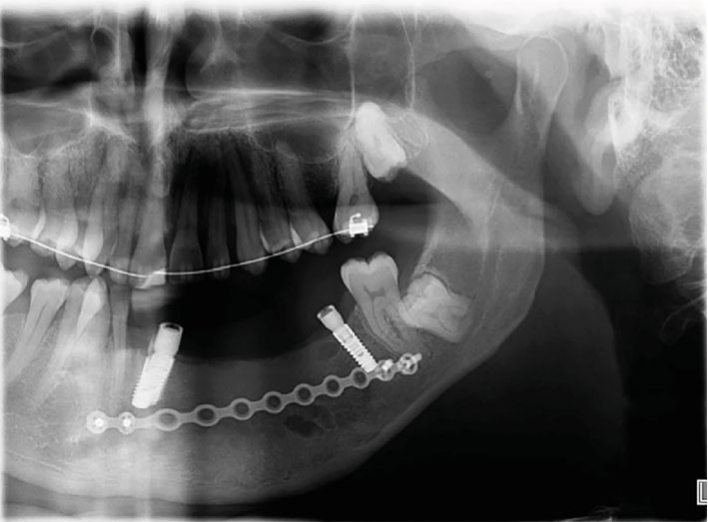
Panoramic view after implant placement-2013.

Due to the deep bite, orthodontic therapy was started in the upper jaw, and in the lower jaw, implant-prosthodontic rehabilitation was planed with minimally invasive procedures because of the lack of a support zone and defect of soft and bone tissues. Regional nerve block anesthesia (4% articaine with epinephrine 1 : 200 000; 3.6 mL) was administered. After carefully raising the mucoperiosteal flap and isolating the mental nerve, the bone crest was expanded at the planned implant sites, with a piezosurgical bone saw (Piezomed, W&H; Austria: power 90%, cooling 80%, “power” operating mode), and two narrow diameter implants (Straumann, Bone Level Roxolid 3.3 × 10 mm, Basel, Switzerland) were placed at positions 32 and 36 (Figures 7 and 8). After implant placement augmentation was performed with xenogenic bone material (Cerabone, Botiss, Germany) and a resorptive native pericardium membrane (Jason membrane 15 × 20 mm, Botiss, Germany), the sutures were removed on the 10th postoperative day at which time the surgical site was healing as expected.

**Figure 9:**
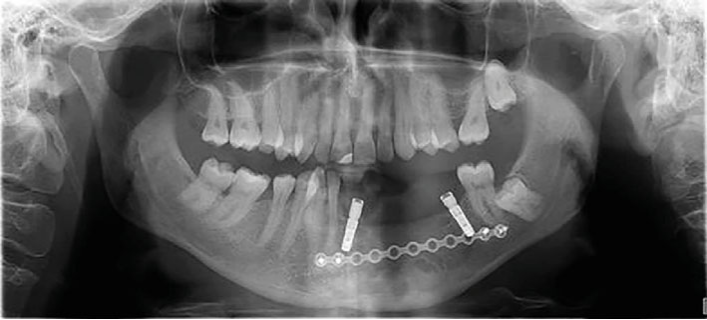
OPG (2020)—9-year follow-up.

**Figure 10:**
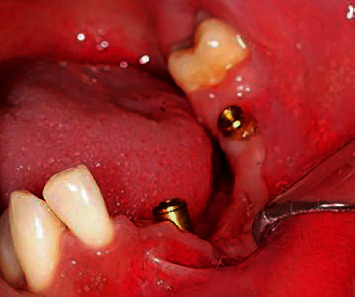
Clinical situation with locators (2020).

**Figure 11:**
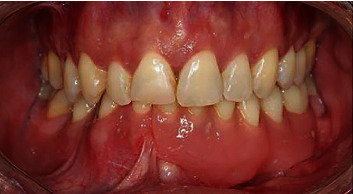
Clinical appearance after 9 years postsurgery and 7 years after implanto-prosthodontic rehabilitation.

Three months after surgery, a cover denture was made with locator connections to compensate for lost teeth and to correct the ratio of soft tissues and facial contours. No tumor recurrence was found on the follow-up OPG and CBCT for 7 years after implant placement and 9 years postresective surgery (Figures 9–11). The patient was subjectively free from functional and aesthetic problems.

## 3. Discussion

According to the literature [16], it is reported that odontogenic myxoma is in 0.5-20% of all odontogenic tumors in adults. There is also a regional difference in the prevalence of these tumors, it is more common in the Caucasian and African populations of those from the far east [17]. Rashid and Bashir [18] stated that odontogenic myxoma mainly occurs in the second and third decade of life and is rarely seen in patients younger than 10 years and older than 50 years, while Takahashi et al. [19] reported that the median age in their study of 12 patients was 41.5 years and 5 were over 50 years old. Our patient in this case report was in accordance with this data regarding its age. The exact predilection of odontogenic myxoma to either the upper or lower jaw is also a matter of discussion. Most authors [20–22] stated that the lesion is more common in the mandible, especially the molar and ramus region, whereas Subramaniam et al. [16] found equal incidence in both jaws in series of 8 cases. Regardless of the jaw, odontogenic myxoma is usually found in relation to a tooth, typically a premolar or molar [23]. Some researchers reported that the lesion is most often found in the mandibular premolar area in series of 37 cases [24]. In the present case, the lesion was in the region of the mandible involving teeth from the canine to the first molar. Odontogenic myxoma often expands the cortical plates in one or more directions, sometimes perforates the cortical surface, and produces a soft feeling on palpation and impression of fluctuance. Due to its absence of a capsule, it can penetrate into marrow space and be very aggressive [25, 26].

Clinically, the presented case showed symptoms that match all of these mentioned signs but without aggressive behavior and the lingual cortical plate was preserved.

Odontogenic myxomas have been diagnosed with many different imaging features like conventional radiography such as panoramic, occlusal, and periapical, or more accurately like cone beam computed tomography/computed tomography (CBCT/CT) and magnetic resonance imaging (MRI) [27, 28]. Wang et al. [29] stated that involved teeth may have resorption or displacement and a combination of resorption and displacement. The patient in this case report did not have either of these, only highly expressed teeth mobility.

Although benign, odontogenic myxoma is invasive into surrounding normal bone, sometimes breaking through its boundaries [5]. This invasiveness has been attributed to the expression of matrix metalloproteinases (MMP) 2 and 9, which degrade the extracellular matrix (ECM) due to its ability to degrade type IV collagen, the major structural component of the basement membrane. These enzymes purportedly cause tumor cells to penetrate the bony trabeculae by acting on the ECM, thus aiding tumor [30]. On gross examination, odontogenic myxoma resected from the presented patient was a nodular heterogeneous mass, grayish-white color, without demarcation from surrounding tissue. This finding was in concordance with Takahashi et al. [19] which stated that this kind of tumor is uncapsulated and poorly demarcated from surrounding tissues while Li et al. [23] reported that in their 25 cases, most of them had minimal capsule.

Microscopically, the tumor has a mucoid-rich ECM, with scattered stellate cells, connective tissue fibers, irregular calcifications, bony trabeculae, sparse capillaries, and scant blood vessels. Nests of odontogenic epithelium are occasionally seen but not essential for diagnosis [5, 13, 23]. The ECM comprises eosinophilic mucoid tissue, which resembles the connective tissue of the umbilical cord. Spindle-shaped or stellate cells with small hyperchromatic nuclei and cytoplasmic processes are interspersed in collagen or reticulin fibers [31]. Cellular atypia is rare, and the presence of mast cells has been reported while fibers are oriented toward the tumor periphery [2]. All of these classical features were seen in our case.

Surgical treatment of odontogenic myxoma is usually invasive, and it depends on the size of the tumor from conservative, e.g., enucleation and surgical curettage, to radical treatment like partial or segmental jaw resection with free flaps. Smaller lesions can be treated only by curettage, but larger lesions need resection due to their infiltration to the surrounding bone. Large maxillary and mandibular bone defects can be reconstructed by fibular and iliac-crest osteomyocutaneous or osteomuscular free flaps [32]. Free flap helps in dental-prosthetic option even if the placement of implant is a challenge.

Lack of its encapsulation and loose of myxoid composition is the main reason of recurrence, so the method of removal is a crucial determinant [28]. According to the recent literature [6, 33], the recurrence rate is high ranging from 10% to 43% with a mean of 25%. Recurrence following incomplete removal usually occurs within 2 years, but also there are the same cases in which recurrence occurred later [6]. White et al. [28] found, reviewing the literature, 9 cases in the literature that provided length of follow-up period ranged from 3 to 84 months (7 years) with an average of 36 months (3 years) and without recurrences in those patients. Following-up in the presented case report was 9 years, and there was no sign of recurrence. The literature showed no consistent recommendations regarding the ideal treatment of this tumor. Saalim et al. [34] reported that there was no significant difference in recurrence between conservative treatment and resection. They recommend that conservative treatment should be considered wherever possible to provide optimal quality of life for the patient.

The reconstruction of the mandible after radical treatment can be made by fixed or removable prostheses retained by a system attached to the implant. In some cases with reconstructed mandible with free flaps, there might be a problem due to inadequate mechanical retention during mastication so an implant-supported fixed dental prosthesis is the best solution for treatment [35]. Meloni et al. [36] reported the advantages of a protocol for rehabilitation consisting of prosthetic-guided implant insertion, a noninvasive surgical approach, and immediate loading on fixed prosthesis in oncologic patients.

Implant-prosthodontic rehabilitation after marginal resection of the mandible requires filling the edentulous space and compensating the loss of hard and soft tissues, therefore presenting a particular challenge to prosthodontists. Removable dental prosthesis is preferred to an implant-supported fixed dental prosthesis to maintain oral hygiene and recurrent examinations and for long-term maintenance [37]. Elsyad et al. [38] showed that locator attachments were associated with high retention and stability after wear simulation with minimal retention loss compared to a Dolder bar. Overdenture improves chewing efficiency, increases maximum bite force, and clearly improves satisfaction [39]. After consultation with the patient, it was decided that a minimally invasive approach to reconstruct the resulting defect will be made. In order to primarily satisfy the function as well as the aesthetics and due to the above-mentioned advantages of the cover prosthesis in oncology patients, the plan was to make denture worn on two implants retained with locator attachments.

## 4. Conclusion

Quality of life in patients after radical surgical treatment of odontogenic myxomas is often compromised by loosing function and aesthetic morbidity. A multidisciplinary approach is the basis for complete implanto-prosthodontic rehabilitation of patients after resective surgical oncology.
